# Characterization of Vascular Disease Risk in Postmenopausal Women and Its Association with Cognitive Performance

**DOI:** 10.1371/journal.pone.0068741

**Published:** 2013-07-17

**Authors:** N. Maritza Dowling, Carey E. Gleason, JoAnn E. Manson, Howard N. Hodis, Virginia M. Miller, Eliot A. Brinton, Genevieve Neal-Perry, M. Nanette Santoro, Marcelle Cedars, Rogerio Lobo, George R. Merriam, Whitney Wharton, Frederick Naftolin, Hugh Taylor, S. Mitchell Harman, Sanjay Asthana

**Affiliations:** 1 Department of Biostatistics and Medical Informatics, School of Medicine and Public Health, University of Wisconsin, Madison, Wisconsin, United States of America; 2 Wisconsin Alzheimer’s Disease Research Center, Madison, Wisconsin, United States of America; 3 Department of Medicine, School of Medicine and Public Health, University of Wisconsin, Madison, Wisconsin, United States of America; 4 Geriatric Research, Education and Clinical Center, William S. Middleton Memorial Veteran’s Hospital, Madison, Wisconsin, United States of America; 5 Department of Medicine, Brigham and Women’s Hospital, Harvard Medical School, Boston, Massachusetts, United States of America; 6 Atherosclerosis Research Unit, University of Southern California, Los Angeles, California, United States of America; 7 Departments of Physiology and Biomedical Engineering and Surgery, Mayo Clinic, Rochester, Minnesota, United States of America; 8 Department of Cardiovascular Genetics, University of Utah, Salt Lake City, Utah, United States of America; 9 Department of Obstetrics and Gynecology and Women’s Health and Dominick Purpura Department of Neuroscience, Albert Einstein College of Medicine, Bronx, New York, United States of America; 10 Department of Obstetrics and Gynecology, University of Colorado School of Medicine, Aurora, Colorado, United States of America; 11 Department of Obstetrics and Gynecology, University of California at San Francisco, San Francisco, California, United States of America; 12 Department of Obstetrics and Gynecology, Columbia University College of Physicians and Surgeons, New York, New York, United States of America; 13 VA Puget Sound Health Care System and Division of Metabolism, Endocrinology, and Nutrition, University of Washington, Seattle, Washington, United States of America; 14 Department of Obstetrics and Gynecology, New York University, New York, New York, United States of America; 15 Department of Obstetrics and Gynecology, Yale University School of Medicine, New Haven, Connecticut, United States of America; 16 Kronos Longevity Research Institute and Phoenix VA Medical Center, Phoenix, Arizona, United States of America; University Heart Center, Germany

## Abstract

**Objectives:**

While global measures of cardiovascular (CV) risk are used to guide prevention and treatment decisions, these estimates fail to account for the considerable interindividual variability in pre-clinical risk status. This study investigated heterogeneity in CV risk factor profiles and its association with demographic, genetic, and cognitive variables.

**Methods:**

A latent profile analysis was applied to data from 727 recently postmenopausal women enrolled in the Kronos Early Estrogen Prevention Study (KEEPS). Women were cognitively healthy, within three years of their last menstrual period, and free of current or past CV disease. Education level, apolipoprotein E ε4 allele (APOE4), ethnicity, and age were modeled as predictors of latent class membership. The association between class membership, characterizing CV risk profiles, and performance on five cognitive factors was examined. A supervised random forest algorithm with a 10-fold cross-validation estimator was used to test accuracy of CV risk classification.

**Results:**

The best-fitting model generated two distinct phenotypic classes of CV risk 62% of women were “low-risk” and 38% “high-risk”. Women classified as low-risk outperformed high-risk women on language and mental flexibility tasks (*p* = 0.008) and a global measure of cognition (*p = *0.029). Women with a college degree or above were more likely to be in the low-risk class (*OR* = 1.595, *p* = 0.044). Older age and a Hispanic ethnicity increased the probability of being at high-risk (*OR* = 1.140, *p* = 0.002; *OR* = 2.622, *p* = 0.012; respectively). The prevalence rate of APOE-ε4 was higher in the high-risk class compared with rates in the low-risk class.

**Conclusion:**

Among recently menopausal women, significant heterogeneity in CV risk is associated with education level, age, ethnicity, and genetic indicators. The model-based latent classes were also associated with cognitive function. These differences may point to phenotypes for CV disease risk. Evaluating the evolution of phenotypes could in turn clarify preclinical disease, and screening and preventive strategies.

ClinicalTrials.gov NCT00154180

## Introduction

Several known risk factors for the development of vascular disease have been linked to not only cardiovascular (CV) disease endpoints, but also to accelerated cognitive decline and prodromal Alzheimer’s Disease (AD) [1]–[5]. Despite public awareness campaigns and the availability of well-established preventive options, CV disease remains one of the leading causes of death for women in the United States [6]–[7]. Menopause-related changes in hormonal profile may potentiate the increased risk in CV disease. Postmenopausal women are at higher risk than age-matched men, possibly due to gonadal failure and reduced gonadal steroid production [8]. Estrogens play a key role in maintaining adequate levels of high-density lipoprotein cholesterol (HDL-C), a positive influential CV health factor and a significant independent predictor of nitric oxide-dependent coronary vasodilation (as measured by flow-mediated dilation) in healthy individuals [9]–[10]. In contrast, elevated plasma levels of low density lipoprotein cholesterol (LDL-C) contributes to endothelial dysfunction and progression of coronary heart disease [11]–[12]. Endothelial dysfunction, often characterized by decreases in production of nitric oxide, is mediated not only by lipid profile, but also by other putative CV risk factors such as elevated glucose and triglyceride levels and high-sensitivity C-reactive protein [13]–[14].

The association between these classical CV disease risk factors and the magnitude of reactive hyperemia in small arteries can be observed in individuals with no history of CV disease and normal lipid profile [15]. Findings further suggest that menopause and aging also are independent risk factors for endothelial dysfunction in normotensive women [16]. Moreover, predisposing vascular disease risk factors such as central obesity, unhealthy diet and physical inactivity may synergistically increase risk for vascular disease (for example, low serum HDL-C, diabetes, and elevated blood pressure). Lower education and income are socio-economic factors often linked to increased risk of vascular disease, especially in women [17]–[18]. Genetic characteristics such as the apolipoprotein E (APOE) gene polymorphism, particularly the presence of an ε4 allele (or APOE4 isoform), are frequently linked, not only to hyperlipoproteinemia, but also to AD and cognitive impairment in non-demented adults [8], [19].

The importance of a multifactorial approach to the evaluation of both traditional and newer markers of vascular risk has been shown to augment the predictive accuracy of global estimates of CV risk [1], [20]. Thus there is an important need for increased understanding of the prevalence of these risk factors as well as their covariation. However, the assessment of a multiplicity of risk factors often involves the use of laboratory values with no “natural” cut-off between “normal” and “abnormal” levels primarily because some of these measurements (e.g., lipid levels) are continuous. Moreover, the threshold or cut-point at which a potential risk factor in the continuum of risk exposure can be considered a “true” risk is a subject of debate [21]–[22]. For instance, specific thresholds for arterial hypertension and hyperlipidaemia are on an arbitrary dichotomy [23]. It is also possible that cut-off criteria for conventional vascular risk factors may in fact vary systematically by gender, race/ethnicity or socio-cultural background. Indeed, global measures of risk based on a graded summation of factors, such as Framingham-based risk scores, have required re-calibration to improve accurate risk estimation in older women [24] and in ethnic minorities [25]. Moreover, the interactive influence of these characteristics may give rise to complex differential effects on health outcomes. Even when the assessment of vascular disease risk yields results within clinically ‘acceptable’ ranges there can be considerable between- and within-individual heterogeneity in the measures considered in the global evaluation of “risk.”

Using a finite mixture modeling approach, [26] this study aimed at elucidating potential phenotypic heterogeneity in risk based on multiple measures of vascular disease risk obtained at baseline from a cohort of postmenopausal women enrolled in the Kronos Early Estrogen Prevention Study (KEEPS) [27]. We hypothesized that 1) co-variation among multiple manifest vascular disease risk factors could be fully explained by a discrete *latent* variable (latent groups) capturing heterogeneity in the sample and 2) latent group or class membership will be associated with demographic, racial/ethnic, genetic, and cognitive function variables.

## Methods

### Sample Description and Setting

KEEPS and KEEPS-Cog studies were reviewed and approved by Institutional Review Boards at all nine enrollment sites and at the University of Wisconsin, the KEEPS Cognitive and Affective (KEEPS-Cog) coordinating site. IRB numbers for KEEPS institutions: The central KEEPS and Phoenix KEEPS (IRB protocol by the Western IRB): STUDY NUM: 1058663 and WIRB PRO NUM: 20040792KEEPS (main study & cognitive substudy) #10-02980 and MDBHAS #11-05383. Brigham and Women’s Hospital (Partners): #2004-P-002144 BWH. Mayo Clinic: 2241-04. Columbia: IRB#: AAAA-8062. Yale: 0409027022. University of Utah: 13257. Einstein/Montefiore: 04-08-213. University of Wisconsin, Madison: H-2005-0059. University of California, San Francisco (UCSF): KEEPS (main study & cognitive substudy) #10-02980. University of Washington: IRB #26702; VAPSHCS IRB #01048.

All participants provided written informed consent to participate in the main KEEPS study and in the KEEPS-cog ancillary study. The ethics committees approved the consent procedure utilized in the study. Enrollment occurred between August 2005 and July 2008 with final visits completed in 2012.

Data for this study were obtained from the multisite KEEPS and KEEPS-Cog substudy. The parent study, KEEPS, was a randomized, blinded, placebo-controlled clinical trial designed to compare the effect of 48 months of treatment with low-dose oral conjugated equine estrogen and transdermal estradiol to placebo on cardiovascular endpoints in recently menopausal women [27]–[28]. The KEEPS-Cog ancillary study aimed to evaluate the potential differential efficacy of the two forms of menopausal hormone therapy (MHT) on cognitive and mood function. Participants were recruited from nine sites across the nation. Exclusion criteria for the trial included the presence of past or current CV or cerebrovascular disease, uncontrolled hypertension, and use of lipid lowering medications. Determinations of “low risk” for CV disease were based on body mass index (BMI), blood pressure, fasting cholesterol and glucose values, tobacco use, and assessment of coronary artery calcification (CAC) measured by computerized tomography (CT). For a more detailed overview of the KEEPS study design, sample enrollment criteria, and randomization and data collection procedures, please refer to the comprehensive descriptions provided in Harman et al. [27] and Miller et al. [29].

The mixture modeling analysis used baseline (pre-randomization) data from 727 postmenopausal women, between the ages of 42 and 58, who were within 3 years of their final menstrual period. **Table 1** presents a summary of the sample characteristics at study entry. In terms of demographic characteristics, the sample was predominantly non-Hispanic white (80.5%), averaged 53 (*SD = *2.6) years of age, and 73.5% reported having obtained a minimum of a college degree. Additionally, of those consenting to DNA testing (N = 596), 26% had at least one APOE ε4 allele; a genetic risk marker often associated with adverse changes in cognitive functioning occurring prematurely during the aging process, [30]–[31] CV disorders in middle age, [32]–[33] and shown to interact with female gender [34]–[35]. Most participants (87%) were entirely free of CAC at baseline. The remaining 13% of the sample had CAC volume scores ranging from 0.015 to 50.

**Table 1:** Selected Demographic and Clinical Characteristics of the Total Sample at Baseline.

Characteristics (*N*, *Mean ± SD, Range; unless otherwise noted*)			
Demographic	*N*	*Mean ± SD)*	*Range*
Age (*years*)	727	52.68**±**2.60)	42 to 58
Years since menopause	725	1.44**±**0.73)	1 to 3
Self-reported race/ethnicity **(** ***N, %*** **)**	(692)		
* Asian or Pacific Islander*		21(3.03)	–
* Black/African American*		54 (7.80)	–
* Non-Hispanic White/Caucasian*		557 (80.49)	–
* Hispanic*		53 (7.70)	
* Other*		7 (1.01)	–
Education **(** ***N, %*** **)**	(717)		
* Grade school*		3 (0.42)	–
* Some high school*		3 (0.42)	–
* High school diploma or GED*		52 (7.25)	–
* Some college or vocational school*		132 (18.41)	–
* College graduate*		293 (40.86)	–
* Some graduate or professional school*		34 (4.74)	–
* Graduate or professional degree*		200 (27.89)	–
Apolipoprotein E ε4 allele (APOE4) **(** ***N, %*** **)**	(596)		–
		156 (26.2)	
**Vascular Disease Risk Factors**			
Body mass index (BMI) (kg/m^2^)	727	26.19**±**4.31	16 to 35
Waist Circumference (cm)	716	83.2**±**15.20	57.2 to 256.5
Total Cholesterol (mg/dL)	727	208.10**±**33.7	122 to 315
Mean systolic blood pressure (sBP) (mm/Hg)	727	117.43**±**14.90	82 to 189
Mean diastolic blood pressure (dBP) (mm/Hg)	727	75.30**±**9.22	50 to 113
Mean arterial blood pressure (MAP) [1]	727	89.62**±**10.31	63.3 to 132.3
Low density lipoprotein-cholesterol (LDL-C) (mg/dL)	727	110.90**±**27.8-	11 to 194
High density lipoprotein-cholesterol (HDL-C) (mg/dL)	727	72.0**±**14.60	24 to 129
Triglycerides (mg/dL)	727	87.00**±**55.90	7.0 to 374
Fasting blood glucose (FBG) (mg/dL)	727	79.60**±**10.00	55 to 126
Total Framingham Point Score (FPS)	727	4.00**±**3.19	−5 to 14
Current tobacco use (***N, %***)	(727)		–
		50 (6.90)	
**Measures of Vascular Disease**			
Coronary artery calcification (CAC) volume score	727	1.33**±**5.18	0 to 50.00
Carotid artery intima-media thickness (CIMT)	727	0.72**±**0.09	0.53 to 1.17
**Clinical - Cognitive Scores**			
** Factor Scores**			
* Global Cognition*	662	0.0**±**0.88	−3.06 to 2.54
* Verbal Learning & Memory*	662	0.0**±**0.87	−2.96 to 2.40
* Auditory Attention & Working Memory*	662	0.0**±**0.75	−2.73 to 2.09
* Visual Attention & Executive Function*	662	0.0**±**0.73	−2.15 to 2.01
* Speeded Language & Mental Flexibility*	662	0.0**±**0.79	−2.50 to 2.50
Mini Mental State Examination (MMSE)	647	29.1**±**1.40	22 to 30

(1) MAP was estimated as 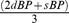.

Selected anthropometric, clinical, serum, and behavioral indicators of vascular disease risk for the total sample at baseline are also summarized in **Table 1**. The mean body mass index (BMI) was 26.19 kg/m^2^ (*SD = *4.31) with close to 50% of the sample with a BMI ranging from 26 to 35 kg/m^2^, which is considered overweight or obese as defined by published standards [36]. Approximately 33% of the sample had a waist circumference above the cut-off score for female central/abdominal obesity (>88 cm) among Caucasians in the United States [37]. Waist circumference measures for the total sample ranged from 57 to 256.5 cm (*M = *83.2, *SD* = 15.2). A relatively small percentage of women in the sample (6.9%) self-identified as current smokers. Although mean values for other vascular disease risk factors shown in **Table 1** were within ‘normal’ reference standards, the lower (e.g., HDL-C) or upper (e.g., total cholesterol, triglycerides, LDL-C) limit for the range of measures, in most risk factors, were slightly beyond the boundaries of ‘clinically desirable levels.

### Laboratory Analyses and Anthropometric Measurements

Seven vascular disease risk variables were used as surrogates for latent class membership in the analysis. These included six *absolute* measures (BMI, carotid artery intima-media thickness (CIMT), LDL-C, fasting blood glucose (FBG), HDL-C, and triglycerides) and a global *average* value of risk based on Framingham point scores (FPS) [38]. All participants underwent venous blood draws in the morning after at least 12 hours of fasting. Blood samples for lipid, glucose, and triglycerides levels measurements were sent to and analyzed by Kronos Science Laboratories (Phoenix, AZ). Blood pressure readings were taken in the morning at least 30 minutes before the blood draws or weight measurement. CIMT was measured by high-resolution B-mode ultrasound [39]. FPS were computed following standard procedures for points assignment and summation described in Wilson et al. [38] from the following six variables: 1) age, 2) systolic blood pressure, 3) diastolic blood pressure, 4) smoking, 5) HDL-C, and 6) total cholesterol. For each of these risk factors, points were assigned according to the level of associated risk.

Height (cm) and weight (kg) measurements were obtained as part of KEEPS health examination protocol and BMI was calculated as weight divided by height squared (kg/m2). **Table 2** summarizes the zero-order correlations for the seven vascular disease risk variables at baseline. As expected, all variables were significantly correlated with at least one other variable in the set; with triglycerides and HDL-C having the highest inverse correlation (***ρ*** = −0.486; *p*<0.002).

**Table 2:** Bivariate Correlations for the Vascular Disease Risk Variables at Baseline.

	Variable	1	2	3	4	5	6	7
**1**	***BMI***	1						
**2**	***CIMT***	0.057	1					
**3**	***LDL-C***	0.157	0.030	1				
**4**	***HDL-C***	−0.336	−0.046	−0.129	1			
**5**	***Triglycerides***	0.330	0.073	0.231	−0.486	1		
**6**	***Fasting Glucose***	0.276	−0.018	0.068	−0.200	0.214	1	
**7**	***FPS***	0.376	0.152	0.399	−0.485	0.458	0.238	1

Correlations greater than the absolute value of *ρ* = 0.12 were significant using a per-test Sidak-adjusted.

*p*<0.002 and a family-wise alpha of 0.05.

BMI = Body mass index; CIMT = carotid artery intima-media thickness; LDL-C = Low-density lipoprotein cholesterol; HDL-C = High-density lipoprotein cholesterol; FPS = Framingham point scores.

### APOE Genotyping

APOE genotype was determined from DNA extracted from venous blood samples obtained from subjects who gave informed consent for genetic analysis. Blood samples were collected in ethylenediaminetetraacetic (EDTA) tubes during participants’ health examination. DNA was amplified by polymerase chain reaction using specific primers for the APOE gene. The DNA was then sequenced and analyzed for genotype using the FinchTV program (Version 1.3; Geospiza, Inc). APOE4, as well as age, race/ethnicity, and education (as an indicator of socio-economic status-SES) were modeled as predictors of latent class membership.

### Assessment of Cognitive Function

As part of the KEEPS-Cog substudy protocol, 662 participants were administered a comprehensive neuropsychological test battery by personnel trained in standardized assessment and scoring procedures. In order to efficiently analyze the cognitive functioning of study participants, a total of 25 test variables were first grouped into cognitive domains based on theoretical considerations. By summarizing our neuropsychological battery into cognitive domains, we limited capitalizing on chance associations in subsequent statistical analyses. These theoretical groupings were then tested iteratively using confirmatory factor analyses (CFA) [40]. We used multiple criteria and recommended thresholds for model selection [41]. These included: 1) comparative fit index (CFI) and Tucker-Lewis index (TLI) greater than 0.95, 2) root mean squared error of approximation (RMSEA) [42] less than 0.05, and 3) the smallest Bayesian information criterion (BIC) [43] value. All models were estimated using maximum likelihood (ML) estimation procedures with standard errors robust to non-normality of observations. The statistics software R, Version 2.15.1 (http://cran.r-project.org/) and the package *lavaan*, [44] Version 0.4–14, were used to fit the CFA models.

**Figure 1:**
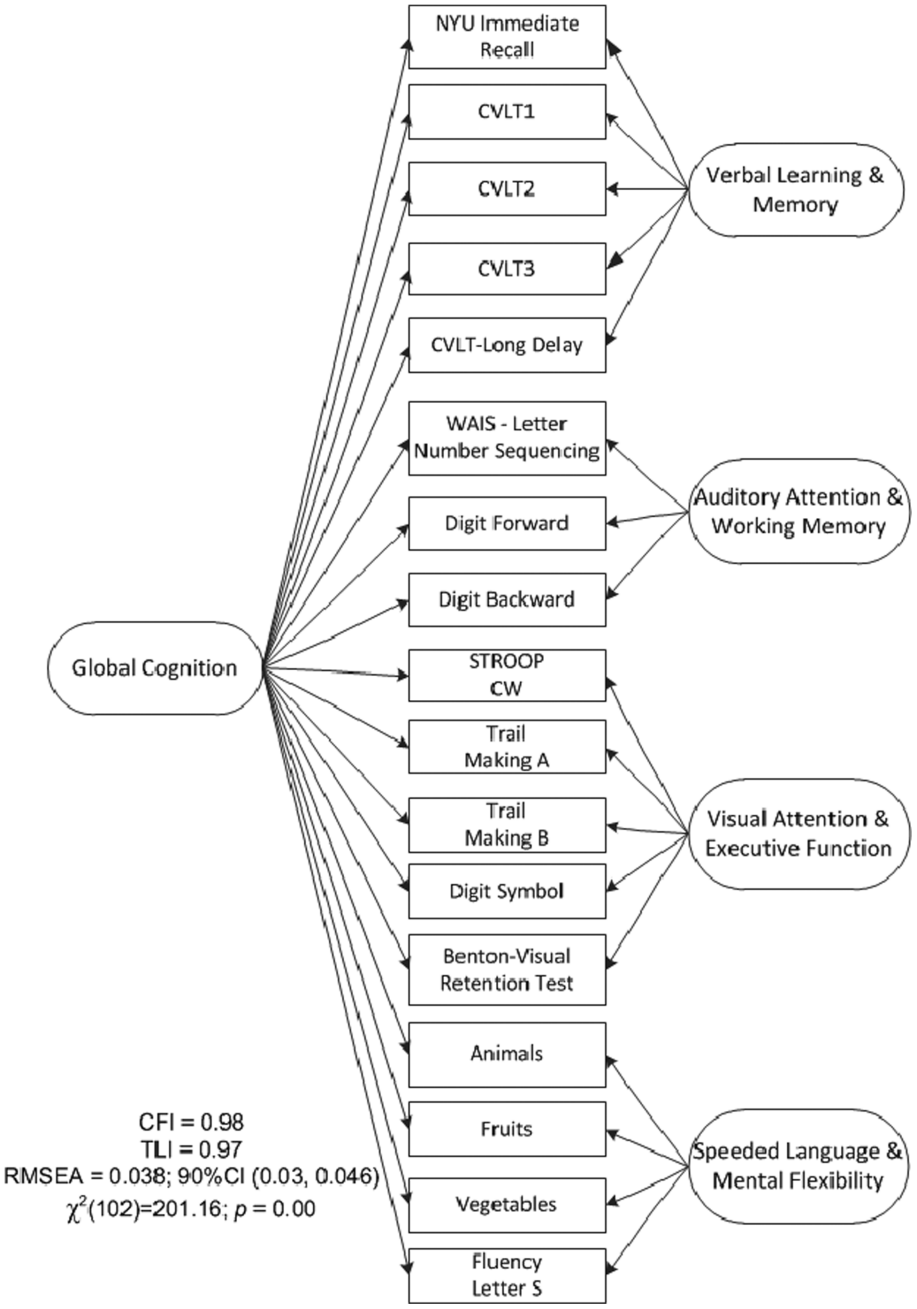
Bi-factor model for the cognitive *baseline* data. Eighteen variables from nine tests were used to estimate the model with a global cognitive factor capturing covariation across all variables and four *independent* secondary factors explaining specific shared covariations *beyond* that shared with other variables.

After testing a series of competing models, a bi-factor structure, [45] including a final set of 18 test variables sharing a common underlying construct, provided the best fit to the data. (For a detailed list of the tests included in the final model and an illustration of the bi-factor model, see **Figure 1** **and Table S1**. **Table S2** presents a summary of the bi-factor solution. The bi-factor model included a single broad, general construct or factor (labeled global cognition) and four specific and distinct factors uncorrelated with and varying independently of the general or global cognition factor. The four specific factors were labeled as 1) verbal learning & memory 2) auditory attention & working memory, 3) visual attention & executive function and 4) speeded language & mental flexibility. Scores on these five factors were modeled as outcomes conditioned upon latent class membership.

### Analytical Approach

To examine sample heterogeneity or clustering, we used multiple vascular disease risk indicators as responses in a finite mixture modeling approach. As mentioned above, the vascular disease indicators included: BMI, HDL-C, LDL-C, triglycerides, FBG, CIMT, and FPS. Since the components of finite mixture densities are modeled as latent classes, the analysis is also known in the literature as latent class cluster analysis or latent profile (LP) analysis [46]–[47]. Within this modeling framework, the clusters or “classes” are not predefined; they are estimated by the model. That is, class membership is unobservable and termed latent. It is reasonable to expect that these indicators will be statistically dependent; for instance, people with elevated triglyceride levels also tend to have low HDL-C levels and other conditions such as obesity and metabolic syndrome. An underlying assumption in the LP analysis is that indicators are associated because the study population is comprised of a mixture of subpopulations or classes [48]. A related underlying principle is that as the number of classes increases the indicators become more “homogeneous” or “locally independent” within class. That is, the mutually exclusive classes derived by the LP model maximize between-group variance and minimize within-group variance.

To determine sample heterogeneity as a function of vascular disease risk, we iteratively examined the plausibility of LP models with one, two, and three-latent class solutions. Models were compared by examining multiple fit criteria: [49] 1) a comparison of an *c*-class solution to an (*c*+1)-class using a Lo-Mendel Rubin likelihood-ratio tests (LRT) [50] with the choice of the most parsimonious model, 2) BIC, and 3) overall model interpretability. We also used relative entropy as a model selection criterion and the requirement of at least 5% of the sample in each class. Relative entropy is a measure of how well the observed indicators predict class membership with values ranging from 0 to 1 and higher numbers indicating better classification. The final decision on the number of classes needed to test the mixture model hypothesis not only took into account model fit indexes, but also an observed separation of classes showing structure and response patterns that were interpretable and meaningful from a theoretical and clinical perspective.

After rigorous model fit and selection procedures for unconditional models (no covariates) using the full pre-randomized sample (*N* = 727), we incorporated four predictors of class membership into the model, in the same step in which the measurement model was run, and re-assessed the composition of the classes. The predictors included: 1) age in years, 2) education level (dichotomized as college degree or higher versus below a college degree), 3) APOE4 status (carriers of the *ε3/4* or the *ε4/4* genotype were categorized as “1;” the absence of the *ε4* allele was categorized as “0”), and 4) racial/ethnic background (categorized as non-Hispanic White, non-Hispanic Black, or Hispanic). Other races/ethnicities were excluded from the analysis performed at this stage, because the number of participants in these groups was not large enough to support meaningful comparisons and ensure some prevalence of the predictor level across classes.

Upon the final model selection, each participant was allocated to the most probable latent class, that is, the class with the highest posterior probability of membership. The posterior probability is a function of the parameters of the LP model, covariates, and the participant’s vascular risk profile.

**Figure 2:**
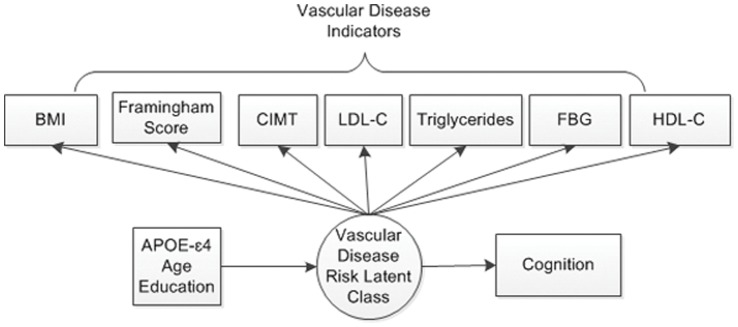
Diagram illustrating the latent profile model. BMI = Body mass index; CIMT = Carotid artery intima-media thickness; LDL-C = Low density lipoprotein-cholesterol; HDL-C = High density lipoprotein-cholesterol; FSG = Fasting blood glucose.

**Figure 3:**
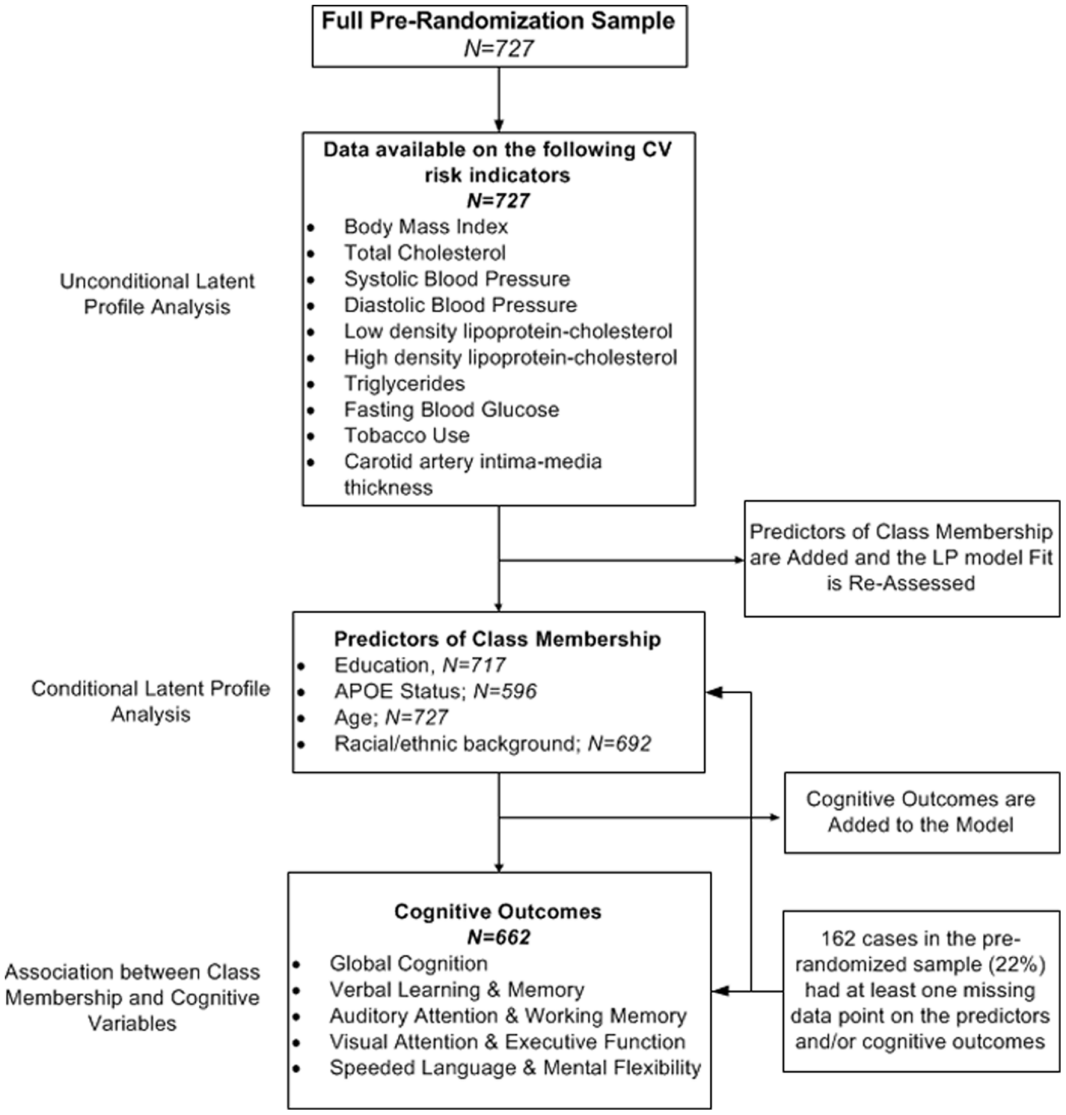
Schematic diagram of the principal steps in the analysis. Models were estimated via full information (direct) maximum likelihood algorithms using all available data.

Finally, estimated latent classes were modeled as explanatory variables of cognition in separate analyses; one for each of the five independent cognitive outcomes. The Wald test, a chi-square analog of an *F*-statistic in analysis of variance, was used to assess the significance of the association between latent classes and cognitive function. (The full model is illustrated in **Figure 2**.) A total of 162 cases in the pre-randomized sample (22%) had at least one missing data point on the predictors and/or cognitive outcomes. (**Figure 3** shows a schematic diagram of the steps in the analysis process.).

The nature of each latent class or “phenotype” was examined by plotting the class-specific estimated mean values against each vascular disease risk variable across classes and inspecting the characteristics of the latent class in terms of CV disease risk profile. To examine the degree to which each class membership could be impacted by demographic and genetic covariates, we used multinomial logistic regression. Odds ratios (ORs) were reported comparing the association between covariates and latent class membership. All LP models were estimated in *MPLUS, Version 6.10,* using full information ML methods via the expectation-maximization (EM) algorithm to handle missing data [51]–[52]. ML estimation was performed under the assumption of missing at random (MAR). [52] We estimated robust standard errors to account for the non-normality of indicator variables. Results yielding a *p*-value less than 0.05 were deemed statistically significant.

### Cross-validation Through Supervised Machine Learning

Bias due to over-fitting is a common criticism of “in-sample” model selection in latent class modeling. We used a supervised random-forest (RF) classification algorithm with a *10*-fold cross-validation estimator [53] to assess overall classification accuracy (or error rate). Latent class membership was modeled as the outcome conditioned upon vascular risk variables. Details of the RF procedure are explained elsewhere [53]–[54]. Briefly, in an attempt to reduce the bias of a “single tree” prediction of classes, *n_tree_* bootstrap samples are drawn from the total data set and for *each* of the samples, a classification tree is grown. The split of each node in the tree is based on a random sample of predictors. New data are predicted by aggregating the *n_tree_* classification trees (i.e., the majority votes for the classification). The accuracy or “error rate” is estimated by predicting the data *not* in the bootstrap sample (generally 1/3 of the sample) using the classification tree obtained *with* the bootstrap sample (2/3 of the sample). All these predictions are aggregated to obtain an estimate of misclassification or error rate. In our analysis, we grew a total of *n_tree_*_ = _1,000 trees. As part of the algorithm, RF estimates variable importance measures for each tree through permutation of variable values. Variable importance is defined as the average increase in error over *all* the trees (mean decrease accuracy) grown in the classifier. The analyses were performed with the randomForest [55] package in *R*, Version 2.15.1 (http://cran.r-project.org/).

## Results

### Latent Profile Analysis

Results for the sequence of unconditional models fitted to the joint distribution of the seven CV disease risk factors indicated that a 2-class LP model adequately fit the data. As shown in **Table 3**, the unconditional 2-class model had the highest classification accuracy or Entropy (0.802), the lowest BIC value, and a reasonable classification of vascular disease risk patterns. The classification accuracy was substantially lower (0.684) for the 3-class model. Despite the rejection of the Lo-Mendell-Rubin LRT test in favor of the 3-class model (marginal *p*-value = 0.04), the separation of classes was less interpretable. Including age, education level, race/ethnicity, and APOE4 as predictors of class membership (that is, the conditional model) improved overall model fit and did not change significantly the prevalence of risk in the two classes. BIC values were lower than those obtained in the unconditional model and the Entropy for the 2-class model increased to 0.811. Additionally, the LRT test indicated that a three-class solution did not represent a significant improvement over the two-class model (*p* = 0.054). Therefore, a 2-class model was chosen as the best fitting model.

**Table 3:** Fit of the Latent Class Profile Models.

Model	No. of ParametersEstimated	Entropy	BIC	Lo-Mendell-RubinAdjusted LRT (p-value)
***Unconditional Model***				
1-Class	15	–	14,444	–
2-Class versus 1-Class	28	0.802	13,821	0.000
3-Class versus 2-Class	30	0.644	13,824	0.040
***Conditional Model***				
1-Class	20	–	11,177	–
2-Class versus 1-Class	33	0.811	10,719	0.000
3-Class versus 2-Class	40	0.694	10,724	0.054

BIC = Bayesian information criterion; LRT = Likelihood ratio test.

**Figure 4:**
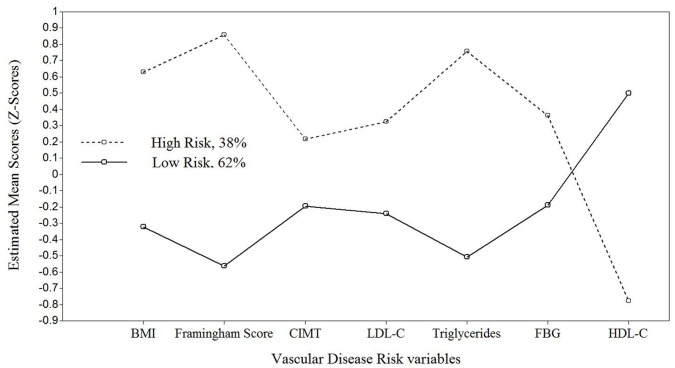
Estimated Mean Vascular Disease Risk for Each Latent Group. BMI = Body mass index; CIMT = Carotid artery intima-media thickness; LDL-C = Low density lipoprotein-cholesterol; HDL-C = High density lipoprotein-cholesterol; FSG = Fasting blood glucose.

The patterns of estimated vascular disease risk measures and posterior probabilities assigned to each individual were used to label the latent classes. The first class was labeled “high-risk” because participants were more likely to have lower values on HDL-C and higher values on triglycerides, BMI, LDL-C, FBG, CIMT, and FPS (see Figure 4). The opposite was observed in the second class, labeled as “low-risk.” That is, participants tended to have higher HDL-C levels and lower triglycerides, LDL-C, FBG, CIMT, and FPS values. The prevalence in the “high-risk” and “low-risk” class was 38% and 62%, respectively. **Table 4** shows the observed and model-estimated means for all the vascular disease risk variables by latent class. Using an independent samples *t*-test, mean differences between groups were highly significant for all vascular disease risk variables (*p-*values<0.001).

**Table 4:** Estimated and Observed Within-Class Means and Standard Errors for Vascular Disease Risk Variables From the Two-Class Model.

Vascular Disease Risk Variables	Estimated Within-Class Means^1^				Observed Within-Class Means				*t*	*p-value*
	*Class 1: Low Risk*		*Class 2: High Risk*		*Class 1: Low Risk*		*Class 2: High Risk*			
	Mean	SE	Mean	SE	Mean	SE	Mean	SE		
BMI	−0.324	0.057	0.630	0.073	24.80	3.88	28.97	3.73	12.57	<0.001
CIMT	−0.194	0.051	0.216	0.082	0.70	0.07	0.74	0.10	5.250	<0.001
LDL-C	−0.240	0.064	0.323	0.069	121.89	28.91	138.35	28.23	6.620	<0.001
HDL-C	0.498	0.055	−0.779	0.065	74.13	15.20	51.22	9.80	−21.751	<0.001
Triglycerides	−0.510	0.051	0.755	0.086	66.73	25.74	129.26	60.24	14.402	<0.001
FSG	−0.188	0.049	0.361	0.083	87.31	8.46	92.73	9.78	6.718	<0.001
FPS	−0.564	0.059	0.857	0.063	2.16	2.25	6.89	2.14	25.926	<0.001
***Latent Prevalence (Marginal Probability)***	62%		38%							

1 The estimated within-class means represent the mean difference between the vascular disease risk score of that particular class compared with the overall mean. Estimated means are based on standardized measures.

BMI = Body mass index; CIMT = Carotid artery intima-media thickness; LDL-C = Low density lipoprotein-cholesterol; HDL-C = High density lipoprotein-cholesterol; FSG = Fasting blood glucose; FPS = Framingham point scores.

### Predictors of Class Membership

Age, education, and race/ethnicity were predictive of class membership (see **Table 5**). Older age and a Hispanic background increased the probability of being in the “high-risk” class (*OR* = 1.140, *p* = 0.002; *OR* = 2.621, *p* = 0.012; respectively). Women with a college degree or above were more likely to be in the “low-risk” class (*OR* = 0.63, *p* = 0.044). The prevalence rate of women with at least one APOE-ε4 allele was also higher in the “high-risk” class compared with rates in the “low-risk” class (*OR* = 1.52). However, APOE4 was not predictive of class membership (*p* = 0.073).

**Table 5:** Conditional Odds Ratios.

Variable		Wald 95%Confidence Limits			
	Odds Ratio	Lower	Upper	p-value	95% CLR
***High Class on***					
*Education (college degree or higher relative to below college degree)*	0.627	0.398	0.987	0.044	2.5
*Hispanic (relative to non-Hispanic White)*	2.621	1.236	5.56	0.012	4.5
*Non- Hispanic Black (relative to non-Hispanic White)*	0.951	0.426	2.122	0.902	5.0
*Age*	1.140	1.05	1.238	0.002	1.2
*APOE4*	1.521	0.961	2.406	0.073	2.5

Abbreviations: CLR = confidence limit ratio.

### Association between Latent Classes and Cognitive Function

The Wald test of parameter constraints yielded a statistically significant association between latent classes and two cognitive factor scores obtained from the bi-factor solution: 1) speeded language & mental flexibility (χ^2^_(*1df*)_ = 6.995; *p* = 0.008) and 2) the general global cognition factor (χ^2^_(*1df*)_ = 4.786; *p* = 0.029). The estimated mean cognitive scores were significantly better in the “low-risk” class for speeded language & flexibility (*M* = 0.068) and global cognition (*M* = 0.772) compared to those obtained in the “high-risk” class (*M* = −0.139; *M* = −0.110, respectively). In a post-hoc analysis, we estimated the effect of posterior probabilities of class membership on cognitive performance across all domains after controlling for group differences in age and education. The relationship between the probability associated with membership in the “high” risk class and performance in speeded language and flexibility tasks remained highly significant (*p* = 0.001). That is, the higher the probability of being in the “high” risk class, the lower the score in speeded language and flexibility. However, differences in global cognition outcomes, as a function of class probabilities and age and education covariates, were attenuated (*p* = 0.06). In both analyses, latent classes were not associated with performance on three specific factors in the bi-factor model, namely, verbal learning & memory, auditory attention & working memory, and visual attention & executive function.

### Cross-validation through Random Forests

**Figure 5:**
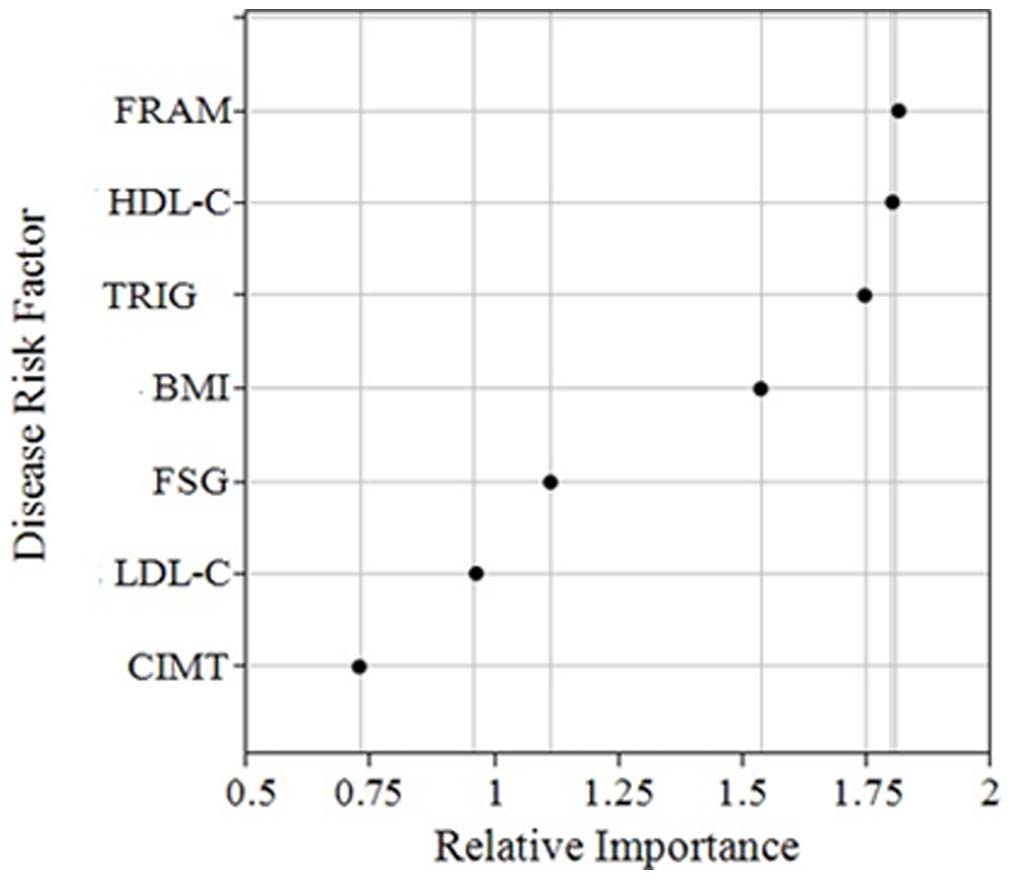
Variable Importance Measures Estimated by Mean Decreased Accuracy. BMI = Body mass index; CIMT = Carotid artery intima-media thickness; LDL-C = Low density lipoprotein-cholesterol; HDL-C = High density lipoprotein-cholesterol; FSG = Fasting blood glucose; FRAM = Framingham Point scores; TRIG = Triglycerides.

We used the classifications obtained through LP analysis as a dependent variable conditioned upon vascular risk and predictor variables to assess the performance of a RF algorithm at predicting class membership. A 10-fold cross-validation estimator was used to assess overall error rate. The RF algorithm yielded an estimated classification accuracy of 96%. That is, the classifier allocated individuals into the “correct” or “LP-estimated class” with approximately 4% error rate. These results support the previously obtained solution and the observed pattern of vascular disease risk measures meaningfully separating individuals into two distinct groups or phenotypes. Interestingly, the weights assigned to each vascular disease risk variable ranking their “importance” as predictors in the RF classifier (see **Figure 5**) corresponded closely to the observed separation of classes. For example, the four variables with the highest importance weights (HDL-C, FPS, triglycerides, and BMI) also produced the highest separation between the latent classes illustrated in **Figure 4**.

## Discussion

A latent profile (LP) analysis, using seven clinically-relevant variables for CV disease risk obtained at baseline from a cohort of recently menopausal women enrolled in the KEEPS study, revealed two distinct classes or phenotypes, depicting low versus high CV risk. The low CV risk group was, as expected, larger, with 62% of the respondents, while the high risk group comprised the remaining 38%. Our results supported the hypothesis that genetic and demographic variables were predictive of the model-identified classes or phenotypes. An interesting finding is that latent class membership was significantly associated with performance in cognitive tasks. That is, individuals in the low CV risk group, on average, obtained significantly higher scores particularly on executive function tasks measuring speeded language and mental flexibility compared to those in the high CV risk group. The speeded language and mental flexibility factor score was composed of tests of letter and category word list generation, which have been frequently used to investigate the semantic fluency deficits related to the progression of AD [56]–[57] and ischemic vascular dementia [58]–[59].

Midlife CV risk factors are well-known non-genetic risk factors for incident AD and cognitive decline. Published studies have also shown a relationship between higher systolic blood pressure or total cholesterol and LDL-C concentrations in midlife and increased risk of cognitive impairment or AD [60]–[62]. For example, Knopman et al. [60] found an association between hypertension and decline in processing speed tasks over a 6-year period. The relationship between BMI and cognitive function appears more complex, possibly varying depending on the location of adiposity. A recent study [63] using baseline data from the seminal Women’s Health Initiative (WHI) hormone trials cohort reported an inverse association between BMI and performance on the Modified Mini-Mental State examination (3MSE); a measure of global cognitive functioning. Interestingly, this association was stronger in women with smaller waist to hip ratio (WHR) (<0.78) and weaker with higher WHR and BMI measurements. These findings suggested a relationship between BMI and cognitive function conditioned upon abdominal obesity in cognitively normal older postmenopausal women. A second study using 4-year follow-up data from women enrolled in WHI Memory Study (WHIMS) [64] found significant interactions between BMI, WHR, and incident cognitive impairment and probable dementia. That is, in women with BMIs between 20 and 29.9 kg/m^2^, central adiposity (WHR≥0.80) was associated with an increased risk of cognitive impairment and probable dementia. Although the mechanisms underlying the complex associations between both indices of obesity and cognitive function are unclear, our study demonstrated the synergistic role of obesity, as measured by BMI, in identifying unobserved group heterogeneity.

The present study suggests that even within a relatively healthy sample of postmenopausal women at “low” CV risk, vascular disease risk factors exhibit important heterogeneity. The LP approach captured cross-sectional group differences in CV disease risk associated with demographic, genetic, and cognitive variables. To the best of our knowledge, this is the first study investigating whether model-based CV disease risk profiles or groups, based on multiple risk criteria, are associated with cognitive function in recently menopausal women. It is possible that the latent “at risk” group identified by this analysis is capturing women at increased risk for the vascular pathway to AD. The use of model-based analytical approaches to identify systematic heterogeneity and complex “within-class” inter-relationships among multiple biomarkers of risk may be more informative than using standard group-based approaches or “total” sample average scores of vascular risk variables. The accuracy and utility of single estimates of CV risk, such as FPS, can be greatly enhanced by considering additional factors that may help explain the considerable individual variability in risk that may exist in the larger population. Absolute risk in the Framingham population for a given set of factors may not be the same as that for all other populations with differing characteristics such as ethnicity. Therefore, the risk assigned by the FPS may miss a large number of individuals destined for CV events. Newer biomarkers such as CIMT and CAC scores, and both predisposing (e.g., BMI, physical inactivity, and abdominal obesity) and conditional (e.g., inflammatory markers and elevated serum triglycerides and lipoprotein) risk factors may potentially modify the magnitude of risk for individuals [65]–[66]. Our findings imply that a mixture-based approach can have potential to study the relatedness of multiple risk variables beyond single risk scores measures representing average values.

Our phenotypes portray a pattern consistent with a large number of studies showing the varying prevalence of risk factors and the underlying rates of CV disease events according to age, education level (as an indicator of SES), and race/ethnicity [67]–[72]. For example, in the Framingham study, women with less than 12 years of education had nearly a four-fold higher risk of developing CV disease than women with higher education level [69], [71]. The same study also reported a higher incidence of CV disease among postmenopausal women (up to the age of 55) than that found in younger pre-menopausal women. The Cardiovascular Health Study, a longitudinal study designed to examine risk factors for coronary artery disease in a large population of 5,201 men and women, reported that heavier weight at age 50 (i.e., a BMI ≥27) had a stronger association with prevalent CV disease in women than current weight at age 65 or older [72].

Present findings are also convergent with studies reporting a higher prevalence of vascular disease risk factors among Hispanics compared to non-Hispanic Whites [67], [73]–[76]. Risk factors observed in these studies included obesity, lower levels of physical activity, incidence of metabolic syndrome, and lipid abnormalities. The observed upward prevalence trends in APOE ε4 carriers among women in the “high risk” class in our study is also in agreement with findings from a large number of studies showing associations between APOE polymorphisms and cardiovascular risk and lipid profile phenotypes [8], [77]–[78]. Other studies have also demonstrated that a decrease in plasma estrogen levels after menopause and APOE may jointly affect lipid and triglyceride levels [79].

A limitation of the current study was the use of a selected number of CV risk factors in the LP analysis. However, these measures differentiated between distinct subclinical phenotypes and suggested a patterning of and unique co-variation in risk associated with cognitive function and demographic features. Further studies could explore the reproducibility of the results in ethnically-varied samples of postmenopausal women. Future longitudinal studies should also investigate the utility of the combination of CV disease risk variables used as surrogates for class membership in this study in predicting not only CV disease, but also cognitive impairment across multiple domains.

We acknowledge that questions remain regarding the complex nature of the interrelationships between vascular risk biomarkers included and not included in the present analysis and their dual prognostic utility for cognitive decline and CV events. Nonetheless, this study highlights the importance of a multifactorial approach to vascular disease risk. The use of an LP framework for the identification of empirically-derived qualitative phenotypes of risk based on a combination of both traditional and newer risk markers can be extremely useful in defining risk scoring systems with better prediction accuracy and clinical relevance for postmenopausal women and ethnic minority groups. Future work designed to evaluate the evolution of phenotypes could in turn contribute to the understanding of preclinical disease and the role of screening and preventive interventions.

## Supporting Information

Table S1 (DOCX)

Table S2 (DOCX)
