# Supplementary material for: Characterization of Vascular Disease Risk in Postmenopausal Women and Its Association with Cognitive Performance
Source: PLoS One. 2013 Jul 17;8(7):e68741. doi: 10.1371/journal.pone.0068741 (PMC3714288; doi:10.1371/journal.pone.0068741)
Supplement: Table S1 — (DOCX) [file pone.0068741.s001.docx]

Table S1

*Neuropsychological Measures Grouped by Conceptual Domain*

| **Domain/Function** | **Test/Scale (18)** |
| --- | --- |
| ***Attention*** | ***Wechsler Adult Intelligence Scale (WAIS-II)*[1]** |
|  | Digit Span Forward |
|  | Digit Span Backward |
|  | Letter-Number Sequencing |
| ***Verbal learning/Memory*** | ***New York University (NYU) Paragraph Recall* Test[2]** |
|  | Immediate Recall Story A |
|  | Immediate Recall Story B |
|  | ***California Verbal Learning Tests (CVLT)2^nd^* Edition[6]** |
|  | CVLT (Learning Trials 1 to 3) |
|  | CVLT (Delayed Recall) |
| ***Processing Speed/Visual Attention/Visual Memory*** | ***Digit Symbol (WAIS-III)*[1]** |
|  | ***Trail Making Test (Part A)*[3]** |
|  | ***Benton Visual Retention* Test[4]** |
| ***Executive Function*** | ***Trail Making Test (Part B)*[3]** |
|  | ***Stroop Color –Word Interference* Test[5]** |
| ***Language/Verbal Fluency*** | ***Verbal* Fluency[7]** |
|  | Animal list generation |
|  | Vegetable list generation |
|  | Fruit list generation |
|  | Verbal Fluency Test: Letter S |

**References**

[1] Wechsler D. (1997). Wechsler Adult Intelligence Scale (3rd ed.). San Antonio: The Psychological Corporation.

[2] Kluger A, Ferris SH, Golomb J, Mittelman MS, Reisberg B. (1999) Neuropsychological prediction of decline to dementia in nondemented elderly. *J Geriatr Psychiatry Neurol*, 12, 168–179.

[3] Reitan RM, Wolfson D. (1993). The Halstead-Reitan Neuropsychological Test Battery: Theory and clinical interpretation (2nd ed.). Tucson: Neuropsychology Press.

[4] Benton A, Hamsher Kd, Varney N, Spreen O. (1983). Contributions to neuropsychological assessment. New York: Oxford University Press; 1983.

[5] Golden C. The Stroop Color and Word Test: A manual for clinical and experimental uses. Chicago: Stoetling; 1978.

[6] Delis DC, Kramer JH, Kaplan E, Ober BA. (2000) California Verbal Learning Test-II. 2nd Edition ed. San Antonio, Texas: The Psychological Corporation.

[7] Spreen O, Strauss E. (1998). *A compendium of neuropsychological tests*. 2nd ed. New York: Oxford University Press.
