# Supplementary material for: Characterization of Vascular Disease Risk in Postmenopausal Women and Its Association with Cognitive Performance
Source: PLoS One. 2013 Jul 17;8(7):e68741. doi: 10.1371/journal.pone.0068741 (PMC3714288; doi:10.1371/journal.pone.0068741)
Supplement: Table S2 — (DOCX) [file pone.0068741.s002.docx]

Table S2

*Estimated Factor Loadings for the Primary and Secondary Factors for the Baseline Bi-Factor Model*

| Test variable | General Factor | Specific Factor | | | |
| --- | --- | --- | --- | --- | --- |
|  |  | Verbal Learning & Memory | Auditory Attention & Working Memory | Visual Attention & Executive Function | Speeded Language & Mental Flexibility |
| *NYU Immediate Recall* | 0.564 | 0.171 |  |  |  |
| *CVLT Trial 1* | 0.349 | 0.498 |  |  |  |
| *CVLT Trial 2* | 0.474 | 0.661 |  |  |  |
| *CVLT Trial 3* | 0.483 | 0.638 |  |  |  |
| *CVLT-Long Delay* | 0.441 | 0.607 |  |  |  |
| *WAIS - Letter Number Sequencing* | 0.655 |  | 0.272 |  |  |
| *Digit Forward* | 0.372 |  | 0.663 |  |  |
| *Digit Backward* | 0.542 |  | 0.431 |  |  |
| *Trails A* | -0.337 |  |  | 0.521 |  |
| *Trails B* | -0.548 |  |  | 0.493 |  |
| *STROOP Color-Word* | 0.508 |  |  | -0.238 |  |
| *Digit Symbol* | 0.465 |  |  | -0.413 |  |
| *Benton-Visual Retention* | 0.497 |  |  | -0.149 |  |
| *Animals* | 0.560 |  |  |  | 0.553 |
| *Fruits* | 0.515 |  |  |  | 0.576 |
| *Vegetables* | 0.497 |  |  |  | 0.614 |
| *Fluency* | 0.544 |  |  |  | 0.347 |

Fit of the Model: CFI=0.98; TLI=0.97; RMSEA=0.038.
